# Development of a Sensor Network System with High Sampling Rate Based on Highly Accurate Simultaneous Synchronization of Clock and Data Acquisition and Experimental Verification †

**DOI:** 10.3390/mi9070325

**Published:** 2018-06-27

**Authors:** Yuji Yamakawa, Yutaro Matsui, Akihito Noda, Masatoshi Ishikawa, Makoto Shimojo

**Affiliations:** 1 Institute of Industrial Science, The University of Tokyo, Tokyo 153-8505, Japan; 2 Graduate School of Information Technology and Science, The University of Tokyo, Tokyo 113-8656, Japan; yutaro_matsui@ipc.i.u-tokyo.ac.jp (Y.M.); Masatoshi_Ishikawa@ipc.i.u-tokyo.ac.jp (M.I.); Makoto_Shimojo@ipc.i.u-tokyo.ac.jp (M.S.); 3 Faculty of Science and Engineering, Nanzan University, Aichi 466-8673, Japan; anoda@nanzan-u.ac.jp

**Keywords:** sensor network system, simultaneous synchronization of clock and data acquisition, high-speed tactile sensor, high-speed vision

## Abstract

In this paper, we develop a new sensor network system with a high sampling rate (over 500 Hz) based on the simultaneous synchronization of clock and data acquisition for integrating the data obtained from various sensors. Hence, we also propose a method for the synchronization of clock and data acquisition in the sensor network system. In the proposed scheme, multiple sensor nodes including PCs are connected via Ethernet for data communication and for clock synchronization. The timing of the data acquisition of each sensor is locally controlled based on the PC’s clock locally provided in the node, and the clocks are globally synchronized over the network. We construct three types of high-speed sensor network systems using the proposed method: the first one is composed of a high-speed tactile sensor node and a high-speed vision node; the second one is composed of a high-speed tactile sensor node and three acceleration sensor nodes; and the last one is composed of a high-speed tactile sensor node, two acceleration sensor nodes, and a gyro sensor node. Through experiments, we verify that the timing error between the sensor nodes for data acquisition is less than 15 μs, which is significantly smaller than the time interval of 2 ms or a shorter sampling time (less than 2 ms). We also confirm the effectiveness of the proposed method and it is expected that the system can be applied to various applications.

## 1. Introduction

### 1.1. Background

The technique for integrating tactile, visual, and other sensor information is an essential and important issue in measurement and control, and such sensor fusion techniques can effectively contribute to engineering fields. To seamlessly integrate the sensor data obtained, a method for data-acquisition synchronization is required, which can fuse the sensor data that are coherent over a time series. Consequently, such a technique would simplify sensor data fusion, because the data with the synchronization are highly consistent with time.

Moreover, in the case where tactile, visual, acceleration, force, angle, and angular velocity sensor data are used for accurately controlling robots, machines, and precision devices, a high sampling rate of data acquisition in the sensor network system is required. Further, from the viewpoints of the recent trends in technology such as the Internet of things, artificial intelligence, and big-data analysis, highly dense data are also strongly required to increase the analysis efficiency and validity of its result. Consequently, the performance of various applications described above can be significantly improved using such a data-acquisition technique with a high sampling rate and the synchronization of data acquisition.

Therefore, the required specification in the sensor network system to achieve an accurate measurement and control is summarized as follows:

1. data acquisition at the same timing in every sensor; and
2. highly dense data.

By performing the first term, a transition of the state (for example, among vision, proximity, and tactile) of a target can be observed, and a switching control according to the state transition is enabled. In addition, in the stereo vision (three-dimensional measurement) for a dynamic object, the synchronization of shutter timings of two cameras is required to achieve a precise measurement. Further, considering an occlusion problem that is a common problem in computer vision, multiple camera network systems are required. In this case, the shutter timings of all cameras are to be synchronized. The necessity of the second term is trivial. Thus, we have developed a high-speed sensor network system of various sensors with a high sampling rate and good data synchronization.

Meanwhile, wireless sensor networks have been actively discussed recently. For example, Yick et al. reviewed various wireless sensor network systems. In particular, they study surveyed three kinds of categories, and summarized the designs, algorithms, protocols, and services in the wireless sensor network systems [1]. D’Andreagiovanni and Nardin discussed the healthcare applications of the wireless sensor network [2]. Natalizio et al. proposed a mathematical model to determine the optimal placement of wireless nodes to maximize the lifetime of data flow [3]. Tsouri et al. suggested the routing protocol for increasing the network lifetime in the wireless body of networks [4]. It is considered that our proposed method can extend to the wireless sensor network in the future, and this research contributes to these fields and applications described previously. In the wireless sensor network, the software method is also required to achieve the specifications above. Because our method can be performed in the software architecture, the method is considered applicable to the wireless sensor network.

### 1.2. Related Works

The proposition for synchronizing the shutter timings of multiple cameras in a vision network system has been discussed. In a typical 30 frames per second (fps) camera network system, Litos et al. suggested a clock-synchronization-based method using the network time protocol (NPT) [5]. However, the accuracy of the synchronization by NPT was approximately in the millisecond order, which is not acceptable for high sampling rates over intervals of millisecond or submillisecond order. In particular, this method cannot be applied to feedback control systems that require real-time response. Hou et al. proposed an illumination-based method for the real-time contactless synchronization of high-speed vision systems [6]; however, the system is not sufficiently flexible for sensor network systems. Further, the method is considered applicable to only the vision network system. Namely, a slight lack of the general-purpose properties exists. Other methods for data synchronization in multisensor network systems were proposed in [7,8,9].

To summarize, conventional sensor network systems do not possess a higher sampling rate, a highly accurate synchronization of data acquisition, and general-purpose properties. Our objectives are to solve these problems and develop a high-speed sensor network system with these properties using our proposed method.

### 1.3. Our Previous Researches

We developed a high-speed visual tracking system at 500 fps, in which two high-speed visions provided the precise time stamps of the acquired image information using clock synchronization errors significantly less than the 2 ms frame interval [10]. With this approach, we achieved the frame synchronization of two high-speed vision systems over Ethernet. In addition, we also performed robust target tracking under occlusion with the high-speed vision system [11].

We applied the tracking system based on the high-speed vision network system to an intelligent transport system (ITS) application [12]. Here, the field of vision was expanded and robust object tracking with a wide range was achieved. In this ITS application, the collision avoidance of vehicles moving at high-speed (assuming a real environment, it corresponds to 100 km/h) was also achieved successfully. Meanwhile, we also confirmed that this task could not be achieved in the normal camera network system (30 fps). Consequently, we verified the effectiveness of the high-speed vision network system through the ITS application.

We conducted vibration observations using the high-speed vision network system including four high-speed vision systems [13]. We also statistically analyzed the experimental results and confirmed the effectiveness of the proposed method. Moreover, we constructed a high-speed vision, a high-speed tactile sensor network system, and a sensor network system with tactile sensor and acceleration sensors to confirm the possibility of the expandability of the high-speed vision network system [14,15].

### 1.4. Robot Control with Sensory Feedback

We have been developing a high-speed robot system beyond human [16,17]. We have also demonstrated high-speed and dynamic robotic manipulations, e.g., the batting robot using high-speed visual feedback [18] and the dynamic cloth folding by dual high-speed robot hands [19]. Recently, we proposed a new robot control concept called “dynamic compensation” to perform high-speed and high-precision positioning for industrial robots, and we developed a robotic module to achieve this [20]. These robots can be achieved using high-speed actuators and high-speed visual feedback with high sampling rate, e.g., 1 kHz.

From the viewpoints of such applications, highly dense data are extremely important as input data to the system. Moreover, in the case that tactile, visual, force, acceleration, and gyro sensors data are used for accurately controlling robots, machines, and precision devices, a high sampling rate of data acquisition in the sensor network system is required. Consequently, the performance of various applications can be improved by using such a data-acquisition technique with a high sampling rate. For instance, the catching robot [21] and the running robot [22] will be significantly improved, because the contact or noncontact state is included in these robotic applications.

Real-time feedback is significantly important for optimally controlling the robots, and the high-speed sensing such as tactile, vision, acceleration, and force sensors performs well for sensory feedback and control. In particular, we consider that advanced techniques such as prediction and estimation cannot be applied to and is not suitable for real-time feedback control. Consequently, more robust robot and machine controls will be executed using the high-speed sensing technique proposed in this research.

### 1.5. Purpose

As discussed before, a high-speed sensor network system with clock and data acquisition synchronization using various kinds of sensors has not been developed. We herein propose a data-acquisition synchronization method for high-speed sensor network systems based on an accurate clock synchronization. In clock synchronization, we adopt the precision time protocol (PTP) [23], which works on Ethernet networks. Because Ethernet is highly flexible in communication hardware, it can be utilized over a considerably wide area. In addition, the expandability of the sensor network system in the case where new sensor nodes are added is extremely high. Because a general analog to digital (AD) converter board can be used to acquire data from tactile sensors, the proposed method can be applied to any analog sensors such as acceleration sensors, gyro sensors, and force sensors. Based on the background, we developed a high-speed tactile, visual, acceleration, and gyro sensor network system to observe and control robots, machines, etc. After developing the high-speed sensor network system with the proposed method, we verified the effectiveness of the proposed methods in three kinds of sensor network systems experimentally.

The characteristics of the system include:

1. good data synchronization (approximately ten microsecond order);
2. high sampling rate (over 500 Hz); and
3. general-purpose property (not requiring special hardware).

In particular, the data synchronization improves data analysis, sensor fusion technique, and hardware (e.g., precise machine, robot, etc.) control. The first and second terms correspond to Specifications 1 and 2, respectively, described in Section 1.1. The third term is considered to be a common required specification.

### 1.6. Applications

As applications of high-speed sensor network system, the following can be considered:

- Robot control, which can achieve switch control using the recognition about the transition of contact/noncontact state, and an intelligent control using sensor fusion with multiple sensors’ data.
- Intelligent transport system (ITS), which can perform collision avoidance, lane change, and fully automated driving control.
- Security and surveillance, which can perform the tracking of a number of humans in a huge facility, and robust target tracking under occlusion.

As other applications, human interface, motion capture, monitoring, three-dimensional shape measurement, inspection, and factory automation are considered. A high-speed sensor network system with the proposed method has various potential applications.

## 2. Method for Clock and Data-Acquisition Synchronization

In this section, we explain the proposed algorithm used for the simultaneous synchronization of clock and data acquisition. First, we describe a method for clock synchronization. Next, we propose a method for data-acquisition synchronization.

**Figure 1:**
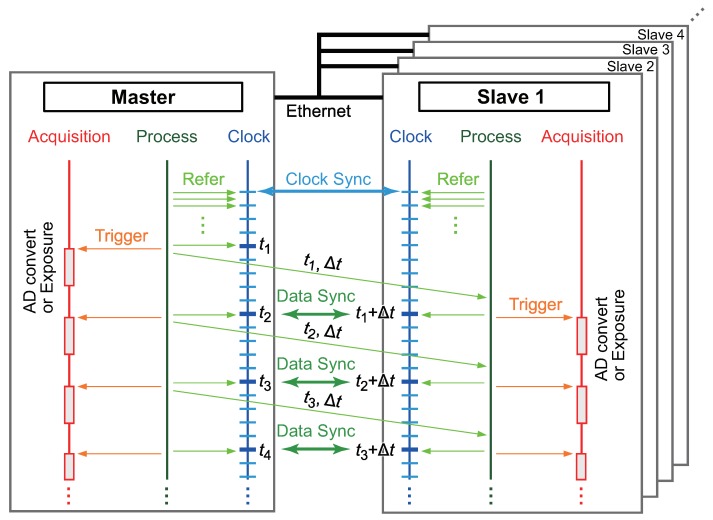
Proposed method for synchronizing data-acquisition timing based on clock synchronization. Adapted from [14].

Figure 1 shows a sequence diagram of the proposed method. For simplicity, a system with a master and a single slave is assumed. Each sensor on the network system is connected to a corresponding PC that can perform the clock and data-acquisition synchronization.

### 2.1. Clock Synchronization

The slave clock is synchronized with the master clock by PTP. A PTP daemon (PTPd) for Linux OS [24] is performed as a background process and is independent of the data-acquisition and processing software. From the statistical data generated by the PTPd, the clock synchronization error is considered to be a few microseconds theoretically. This is an acceptable error for the 500 Hz or higher sampling rate experimental system. The accuracy of the clock synchronization is evaluated in Section 4.1.

### 2.2. Data-Acquisition Synchronization

In this section, we explain our proposed method for synchronizing data acquisition in the sensor network system based on the clock synchronization with the PTPd. The data acquisition from a tactile sensor/acceleration sensor/gyro sensor through the AD board and the image acquisition of a vision system are controlled based only on the local clock, and we assume that these operations are not affected by network delays. The clocks of the master and the slave are globally synchronized over Ethernet, as described in the previous section. The clock synchronization error is significantly smaller than the network delays and the sampling rate. Owing to the significantly small synchronization error, the master and the slave refer to virtually the same clock with negligible delays in the processors.

The acquisition trigger at the slave is dynamically controlled based on the acquisition time stamp and sampling time information sent from the master, as described below.

1. When the first acquisition triggers, the master refers to the clock and sends the time stamp of the trigger, $t_{1}$, and the sampling time, Δt, to the slave via Ethernet.
2. The sent data arrive at the slave after a network delay.
3. The slave calculates the next acquisition timing $t_{2}$ as $t_{2}=t_{1}+\Delta t$, and triggers the acquisition at time $t=t_{2}$. The master also triggers the acquisition at $t=t_{2}$.

Thus, on the condition that the network delay is less than Δt, the acquisition trigger in the slave is synchronized with the acquisition trigger in the master. Consequently, data acquisition can be achieved by this scheme. It is noteworthy that Δt can also be calculated in the slave using two adjacent time stamps previously received, e.g., $\Delta t=t_{2}-t_{1}$.

The proposed method can be applied to multislave systems without any modification. Multislave clocks can be synchronized to the master clock by the PTP. After the clock synchronization, the information regarding the acquisition time stamps on the master and the sampling rate can be sent from the master to all slaves. Based on the synchronized clock and the provided master’s data-acquisition information, all the slaves can execute the proposed method regarding the data-acquisition synchronization. The effectiveness of the data-acquisition synchronization is confirmed in Section 4.2, Section 4.3 and Section 4.4.

## 3. Sensor Network System with High Sampling Rate Based on Highly Accurate Simultaneous Synchronization of Clock and Data Acquisition

The high-speed sensor network system is composed of a high-speed tactile sensor node, a high-speed vision node, a general-purpose acceleration sensor node, a general-purpose gyro sensor node, and processing PCs connected to the sensor nodes. The details of each sensor are explained in the following.

### 3.1. High-Speed Tactile Sensor Node

A high-speed tactile sensor developed in [25,26] was used for acquiring the tactile information (force and two-dimensional contact position). This tactile sensor is composed of a three-layered structure: the two outer layers are of electrically conductive films, and the inner layer is of pressure-conductive rubber. This rubber can change its resistance in the thickness direction depending on the distributed pressure. Consequently, the center position of a two-dimensional distributed load and its total load can be measured. This tactile sensor also possesses a high responsiveness of 1 ms. Thus, this high-speed tactile sensor is considered appropriate for our research.

To obtain the data from the tactile sensor and to handle the data in the processing PC, we used an AD board (PEX-321012 produced by Interface Corporation, Japan; 12-bit, 1 MSPS (Mega-Sample Per Second)). The AD board has a high sampling rate and a high resolution. Therefore, this AD board is also considered suitable to construct the high-speed sensor network system with the proposed method.

### 3.2. High-Speed Vision Node

A high-speed vision system consisting of a high-speed camera (EoSens MC1362 produced by Mikrotron, Unterschleißheim, Germany) and a frame grabber board (microEnable IV-AD4-CL produced by Silicon Software) on a PC was used for performing the object tracking and the measurement of the object position. The camera resolution was set as 640 × 512 pixels in the experiment. The camera shutter was triggered by the frame grabber board, and the trigger was controlled by software on the PC.

In the high-speed vision system, object tracking was performed as follows:

1. The background image was acquired previously. In the tracking method, the target image was extracted by background subtraction.
2. Binarization for the obtained image was executed, and the image centroid $\left(C_{x},C_{y}\right)$ of the target was calculated based on the image moments given by
  (2) $$\begin{array}{ccc} C_{x} & = & \frac{m_{1,0}}{m_{0,0}}, \end{array}$$
  (1) $$\begin{array}{ccc} C_{y} & = & \frac{m_{0,1}}{m_{0,0}}, \end{array}$$
  where $m_{i,j}$ is the (i,j)-th order image moments defined as
  (3) $$m_{i,j}=\sum_{x}\sum_{y}x^{i}y^{j}I\left(x,y\right),$$
  where I(x,y) equals the pixel value at (x,y). $m_{0,0}$ is the zero-order image moment, and $m_{1,0}$ and $m_{0,1}$ are the first-order image moments in *x* and *y* directions, respectively.
3. Once the object image was acquired and the centroid $\left(C_{x},C_{y}\right)$ of the object was measured, a region of interest (ROI) was controlled around the centroid. The ROI size was set as 200 × 200 pixels to reduce the computational load in the experiments.

By repeating these sequences, the high-speed object tracking and the position calculation of the object were performed successfully.

### 3.3. General-Purpose Acceleration Sensor Node

Acceleration sensors were also used. The specifications of the acceleration sensor (352C23 produced by PCB Piezotronics Inc., Depew, NY, USA) are as follows: the resolution is 5 mV/g, the sampling rate ranges from 2 to 10 kHz, and the acceleration sensor can obtain a one-axis acceleration. To obtain acceleration in three dimensions, three acceleration sensors were used in the direction orthogonal to each other. To obtain the data from the acceleration sensor similarly as the high-speed tactile sensor node, we used the AD board (PEX-321012; 12-bit, 1 MSPS), which is the same as the case in the high-speed tactile sensor node.

### 3.4. General-Purpose Gyro Sensor Node

A gyro sensor was also used. The specification of the gyro sensor (603-24K produced by TE connectivity) is as follows: the resolution is 0.083 mV/°/s, and the frequency response is 0–2 kHz. To obtain the data from the gyro sensor similarly as the acceleration sensor node, we also used the AD board (PEX-321012; 12-bit, 1 MSPS).

### 3.5. Overall System

The PCs connecting to the sensor nodes are communicated via 1-Gbps Ethernet in our system. Subsequently, the PTPd was executed to synchronize the clocks of the PCs in the system, and the data-acquisition synchronization was performed using the proposed method explained in Section 2.

## 4. Experimental Verification

In this section, we verify the effectiveness of the developed sensor network system with the proposed synchronization of clock and data acquisition. The following five experiments were conducted:

1. Evaluation of clock synchronization (Section 4.1);
2. Experiment 1 in high-speed tactile and vision sensor network system (Section 4.2.1);
3. Experiment 2 in high-speed tactile and vision sensor network system (Section 4.2.2);
4. Experiments in tactile and acceleration network sensor system with various sampling times (Section 4.3); and
5. Experiments in tactile, gyro, and acceleration sensor network system (Section 4.4).

### 4.1. Accuracy Evaluation of Clock Synchronization

**Figure 2:**
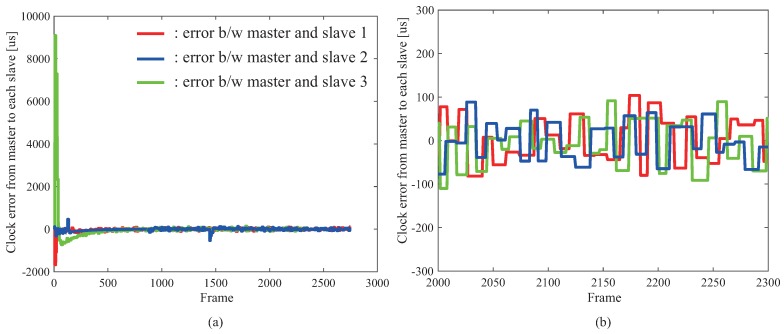
Clock synchronization: (**a**) shows overall result and (**b**) shows enlarged result.

Figure 2 shows the evaluation results of the clock synchronization and depicts an error of clock synchronization between the master and the slaves. In this experiment, the system consists of four PCs, and three errors of clock synchronization between the master and the slaves 1/2/3 were examined. Figure 2a,b illustrates the error from the starting point of the clock synchronization and the error at the steady state, respectively.

From the result shown in Figure 2, because the error of the clock synchronization was approximately 0.05 ms, we confirmed that the clock synchronization with sufficiently high accuracy for high-speed sampling rate was achieved. It can be considered that the high-speed sensing with the sampling rates of 0.2, 0.5, and 1 ms can be achieved. The feasibility of the high-speed sampling rates of 0.2, 0.5, and 1 ms is verified in Section 4.3. The accuracy of the clock synchronization is considered to be improved by tuning the parameters inside PTP.

### 4.2. Result of High-Speed Tactile and Vision Sensor Network System

**Figure 3:**
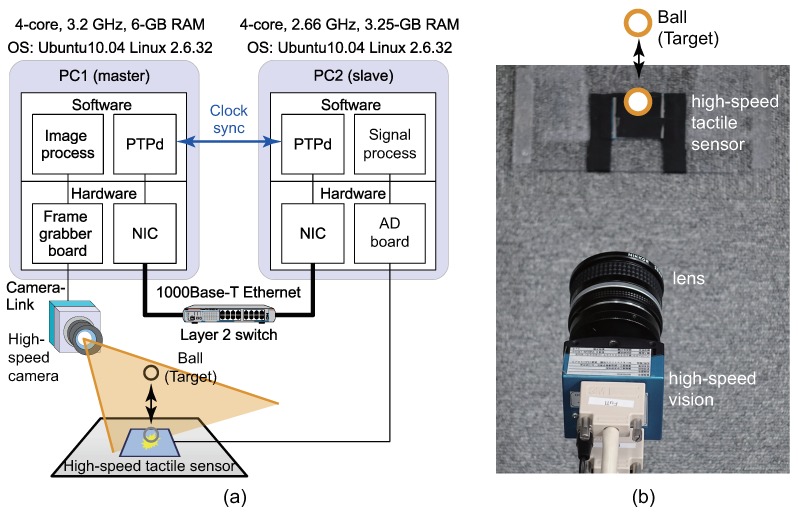
Overview of experimental system setup: (**a**) shows system structure and (**b**) shows experimental environment. Adapted from [14].

To demonstrate that the data-acquisition synchronization in the sensor network system is successfully achieved, we developed a high-speed tactile and vision sensor network system. Figure 3 shows the system structure of tactile and vision sensor network and the photograph of the experimental setup. In the experiment, a ball is moved above or on a table, as shown in Figure 3.

In the tactile sensor node, when the force acted on the tactile sensor, the sensor output corresponding to the force was generated. Subsequently, the AD board converted the sensor output to digital values.

In the vision node, the ball tracking using the method described in Section 3.2 was performed in real time, and the highly accurate position of the ball was obtained by the high-speed vision system within 2 ms.

For this sensor network system, we conducted two experiments as follows:

- Experiment 1: A ball is moved above or on a table by a human. If the ball is above the table, the output of the tactile sensor is zero and the ball position is measured by the vision. Meanwhile, if the ball is on the table, the output of the tactile sensor is obtained and the ball position is also measured by the vision.
- Experiment 2: A ball bounces on a table. If the ball touches the table, the impact force is obtained by the tactile sensor. Otherwise, the force is not obtained by the tactile sensor. In both cases, the ball position can be obtained by the vision.

#### 4.2.1. Experiment 1 [14]

**Figure 4:**
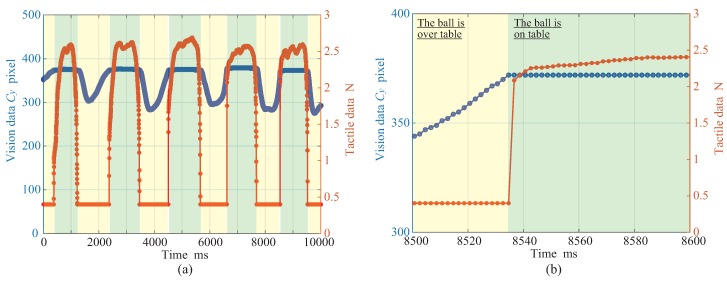
Result of Experiment 1: (**a**) shows tracking data and force data, and (**b**) shows enlarged result. Adapted from [14].

Figure 4a,b illustrates the time series of the force and the ball position obtained by the tactile and the vision sensors, and the enlarged result of Figure 4a, respectively. In the vision data, only the centroid $C_{y}$ of the ball position is plotted because the ball is moved up and down. From the experimental result shown in Figure 4, we confirmed that the highly accurate data-acquisition synchronization in the network system was achieved successfully.

**Figure 5:**
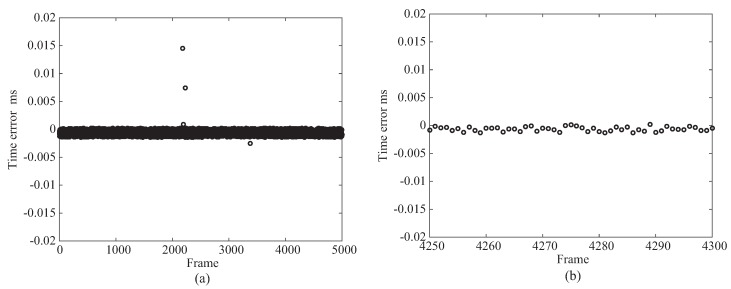
Time error for data-acquisition in Experiment 1: (**a**) shows overall result and (**b**) shows enlarged result. Adapted from [14].

Figure 5 depicts the time error of the data acquisition between the tactile sensor and the vision sensor. From the time error between each data acquisition shown in Figure 5, it could be suppressed to 15 μs at the maximum; thus, we confirmed that the data-acquisition synchronization can also be performed successfully with sufficient accuracy.

#### 4.2.2. Experiment 2

**Figure 6:**
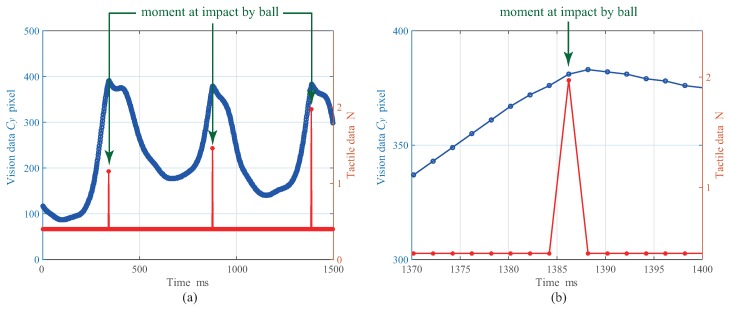
Result of Experiment 2: (**a**) shows tracking data and force data, and (**b**) shows enlarged result.

Figure 6 also shows the time series of the force and the ball position obtained by the tactile and the vision sensors. From the experimental result, we also confirmed that a precise data-acquisition synchronization across the network was performed successfully. The time error between each data acquisition is the same as the result of Experiment 1. In addition, the developed high-speed sensor network system can be applied to an instant phenomenon such as an impact.

Because of the performance of the vision system, the sampling rate in this system is limited to 2 ms. Therefore, we removed the vision system, and we constructed a sensor network system using only an analog sensor, such as the tactile sensor and acceleration sensors, with a sampling rate higher than 2 ms, as shown in the next section. Subsequently, we evaluated the performance of the sensor network system.

### 4.3. Result in High-Speed Tactile and Acceleration Sensor Network System

**Figure 7:**
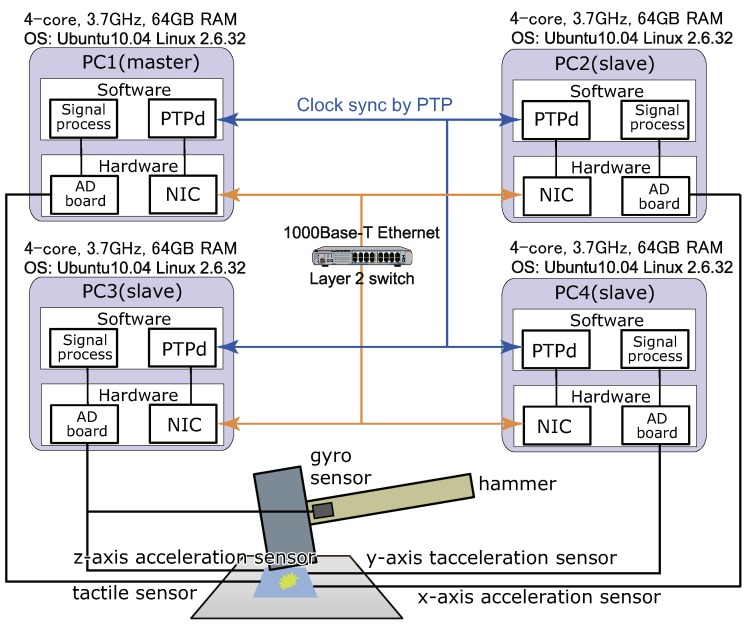
Experimental set-up using various sensors. Adapted from [15].

In this section, we constructed the high-speed tactile and acceleration sensor network system, as shown in Figure 7. Further, we verified the effectiveness of the proposed method in a sensor network system using a single tactile sensor node and three acceleration sensor nodes. In addition, even if the sampling times (1 ms, 0.5 ms and 0.2 ms) are shortened, we confirmed that the system operates similarly as the network system with two sensor nodes. In the experiment, a human impacts the board attached to the tactile sensor and the acceleration sensors by a hammer.

The outputs of the tactile sensor node and the acceleration sensor nodes can be obtained by the same method as described in Section 4.2. In the acceleration sensor nodes, when the force at which the hammer was impacted against the table, the output of the acceleration sensor according to the impact was generated. Subsequently, the AD board converted the output to digital values such that the acceleration data could also be handled on a PC.

**Figure 8:**
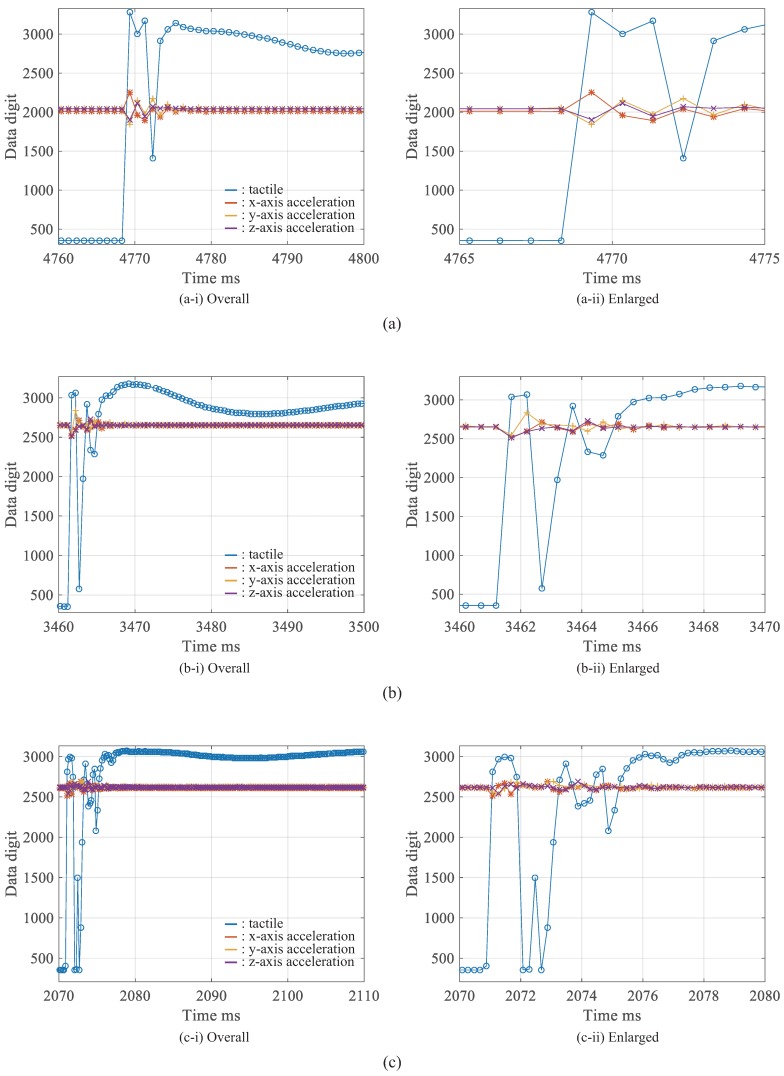
Experimental results with various sampling rates: (**a**) sampling time is 1 ms, (**b**) sampling time is 0.5 ms, and (**c**) sampling time is 0.2 ms.

Figure 8a–c shows experimental results with sampling times of 1 ms, 0.5 ms, and 0.2 ms, respectively. In this experiment, one tactile sensor’s data and three acceleration sensors’ data can be obtained. These sampling times mean the sampling rates of 1, 2, and 5 kHz, respectively. We consider that these sampling rates are high and can be applied to the real-time feedback control systems. It is noteworthy that the validity of the output value is not important in this experiment, but the synchronization of the timing of the data acquisition from various sensors is important in this research. The results show that the data-acquisition synchronization is successful even with a sampling time of 0.2 ms. Furthermore, it has been demonstrated that it can also be applied to a sensor network system using various sensors. Therefore, it is considered that the same result can be obtained for sensor network systems other than this combination. Additionally, it is considered that the performance of the system can be applied to high-speed robots and precise machines.

Moreover, we confirmed from the experimental results that the error of the time stamp was approximately 0.06 μs on average and the standard deviation was approximately 0.3 μs.

### 4.4. Result in High-Speed Tactile, Acceleration, and Gyro Sensor Network System

**Figure 9:**
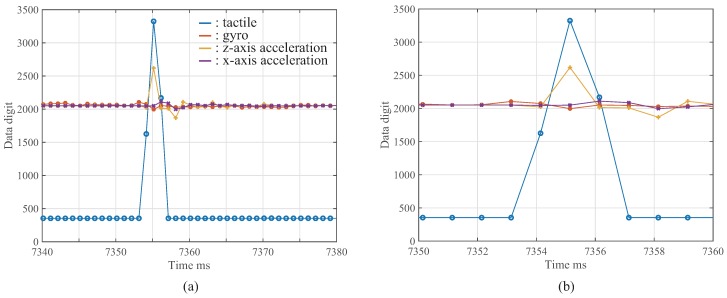
Experimental results with various sensors at 1 kHz: (**a**) shows overall result and (**b**) shows enlarged result.

We constructed a high-speed tactile, acceleration, and gyro sensor network system. Figure 9 shows the experimental result obtained by one tactile sensor, one gyro sensor, and two acceleration sensors. Figure 9 shows that the data-acquisition synchronization with the proposed method was achieved successfully in the developed sensor network system using the various sensors. Consequently, the proposed method can also be applied to the high-speed sensor network system using specific sensors based on the data-acquisition synchronization.

## 5. Conclusions

### 5.1. Summary

In this research, we proposed a method for the data-acquisition synchronization of a high-speed sensor network system using sensors, such as tactile, vision, acceleration, and gyro sensors. In the proposed method, each sensor node refers to its own local clock, and all of the clocks in the network are globally synchronized by PTP. The global clock synchronization can be performed with an error of ten microseconds or less. The error is considered to be significantly smaller than the sampling time (less than 2 ms) for data acquisition. Subsequently, we suggested a method of data-acquisition synchronization based on the clock synchronization. The timing error between the sensor nodes for data acquisition was also evaluated, and we confirmed the precise synchronization of data acquisition with errors less than 15 μs. Because it is considered that this synchronization error is significantly smaller than the sampling time, we conclude that the proposed method is effective for data-acquisition synchronization in a sensor network system.

Moreover, the higher sampling rate (>1 kHz) in the developed sensor network system consisting of a tactile sensor, acceleration sensors, and a gyro sensor can be achieved; additionally, we verified the performance of the sensor network system experimentally. The features of the developed high-speed sensor network system are the following: good data synchronization, high-speed sampling rate, and general-purpose property. In particular, the data synchronization contributes to improving the performance of some applications.

### 5.2. Future Work

For more complicated networks in which many sensor nodes and network switches exist, the network delay will increase, causing the synchronization performance to be degraded, and the delay may exceed the sampling time. Thus, future work will include the evaluation of the limited numbers of network sensor nodes and switches. Subsequently, we will develop a high-speed sensor network system based on a hierarchical structure for achieving a large-scale sensor network system with real-time and data-acquisition synchronization to solve this problem.

For concrete applications described in Section 1.5, our proposed sensor network system with high sampling rate based on the accurate simultaneous synchronization of clock and data acquisition will be applied to robot control with real-time sensory feedback, ITS using vision and other sensor networks, and security and surveillance based on the sensor network system.

Moreover, we plan to extend our sensor network system to a wireless sensor network system using RT-WiFi [27,28]. RT-WiFi technology was proposed for wireless cyber-physical control systems, and the effectiveness of RT-WiFi technology was also confirmed. Because high-speed data communication in wireless network systems can be performed with RT-WiFi technology, we consider that the proposed method (clock synchronization and data-acquisition synchronization) can be applied to RT-WiFi technology.
